# Books and Authors

**Published:** 1909-12

**Authors:** 


### BOOKS AND AUTHORS.

**Essays Concerning the Influence of Visual Function,** pathologic and physiologic, upon the health of patients. By **George M. Gould,** M.D. Volume VI. P. Blakiston's Son & Co., Philadelphia. 1909.

The sixth volume of biographic clinics is just published, thus terminating a series begun by Dr. Gould, in 1903. In these books the author has sought to confirm his theory that defective vision and eye-strain have been factors in the past, which have affected the lives and even the characters of many eminent people. As the skilful physician can, by the study of a portrait, diagnosticate the existence of arteriosclerosis in the subject, so, by the application of accepted modern facts to earlier cases, Dr. Gould has shown that much which has evoked criticism and censure has been due, in many cases, to physiologic causes that may now be clearly determined. The distinguished author here enforces again in his unique manner, his former thesis regarding the far reaching influence of eye-strain. To his former investigations of earlier typical cases he has added in this volume only a study of the peculiarities of Dean Swift, and a second chapter upon Wagner.

In these essays Dr. Gould presents conclusions derived from his own practice, and cites expert "test cases" which cover the entire field of his work. In every instance his conclusions are presented with clearness and acuteness which go to establish the views which he sets forth, and they are confirmed throughout by a system of refraction of remarkable skill and ingenuity, which make them worthy of close examination by those interested in medical research. Dr. Gould has never been satisfied with mere office practice, and has devoted much time to literary work. His contributions to his favorite science, extending over twenty years, have been numerous and have appeared with great rapidity.

The essays which compose the volume prove the author to be an acute and enthusiastic scholar, whose supreme desire has been that the results of his observations may be widely known, so that all classes may be benefited by the recognition of what he believes to be a vital factor in modern medical science. A glance at the headings of the chapters shows how the author has reviewed the whole field of his work, by selecting from among his cases the most significant and illustrative ones. Eye-strain, its cause and its effects, are studied by means of living patients in the chapter on "Brief Biographic Clinics, Etc.;" a "Remarkable Case of Epilepsy caused by Eye-Strain" is given in full in order to supplement several papers previously published on this subject, while one of the most tragic results of eye-strain—namely, suicide, is discussed with judicial care.

The chapter entitled "The Myth and Mystery of Ménière's Disease" and "Ophthalmovascular Choke" deal with new subjects and give the results of the author's most recent investigations. In the former the old mystery which formed the basis of the theory regarding Ménière's disease is cleared away. It is disproved as a typical disease, and its symptoms are traced to the results of eye-strain alone. "It is simply migraine or sick-headache with many symptoms ignored, and three of the secondary or incidental effects of migraine over-emphasized" is the argument with which the essay closes.

"Ophthalmovascular Choke" is a name found for a new disease of the eye, usually discernible by means of the ophthalmoscope, and which consists in a congestion of the veins and insufficient blood supply of the retina. This condition causes a fading of the image, or the inability of the eye to retain an image for more than a short period of time (usually for several seconds only), after which the sensation fails and the image disappears or fades away.

Dr. Gould has always looked upon his work in its philanthropic as well as its scientific relations, and is emphatic in laying great stress on the necessity of carefully studying the individuality of every patient. The chapter on the "Varieties of Medical and Ophthalmic Blunders" which may be made by the ignorant practitioner is a most earnest plea that the good of the patient may be foremost in the mind of the doctor. Humanity is more important than science, and the patient must never be sacrificed to the so-called advancement of learning,—such is his doctrine.

In conclusion we may say that this volume is unquestionably an advance upon Dr. Gould's previous discussions, and is confirmatory of many of the positions which he had earlier assumed, and although in his "Appeal for the Sake of Man and of Medicine," he may at times appear controversial in his statements of facts, even severe in his condemnation of the ignorant or self-interested members of his profession, he is thoroughly in earnest and merits the fullest recognition for his profound and valuable discoveries in his chosen field.

**The Practical Medicine Series.** Comprising ten volumes on the year's progress in medicine and surgery, under the general charge of **Gustavus P. Head,** M.D., Professor Laryngology and Rhinology at the Chicago Postgraduate Medical School. Vol. VI. General Medicine. Edited by **Frank Billings** and **J. H. Salisbury,** Chicago. Series, 1909. Chicago: The Year Book Publishers. (Price, $1.50.)

The topic dealt with in this volume, general medicine, is arranged as before by Frank Billings and J. H. Salisbury. The subjects are, the acute infectious diseases of the mouth, of the esophagus, of the stomach, of the intestines, of the liver, of the pancreas, and of the peritoneum. The subdivisions of each general subject embrace almost every phase of disease that invades the human system, and each one is thoroughly handled, thus giving the general practitioner, the latest points concerning diagnosis and treatment. Diseases of the stomach are given much space as are also diseases of the intestines. Viceroptosis receives due consideration and a number of illustrations serve to demonstrate this curious freak of some of the organs, especially the stomach. This is one of the best volumes on general medicine lately issued.

---

**The Principles of Bacteriology.** A Practical Manual for Students and Physicians. By **A. C. Abbott,** M.D., Professor of Hygiene, University of Pennsylvania. Eighth edition. 12mo, 631 pages, with 100 illustrations, 26 in colors. Lea & Febiger, Philadelphia and New York, 1909. (Cloth, $2.75, net.)

When a book of this character has passed to its eighth edition, it may be taken for granted that almost the last word concerning it has been said, either of blame or praise. Certainly, if it had not met the requirements of the period, it would not now be asking for favor; had it even been damned with faint praise, it is doubtful whether it would have seen half the number of editions that have been posted to its credit.

It may be said, however, that as some time has elapsed since the previous edition during which many advances in bacteriology have occurred, it is fitting that a new edition should now be made setting forth all improvements, new methods of work and everything of known value in the science. This book contains all these and much more not now necessary to mention. It is not only a safe guide for the beginner, but useful to the practitioner.

**International Clinics.** A Quarterly of Illustrated Clinical Lectures and especially prepared articles on Treatment, Medicine, Surgery, Neurology, Pediatrics, Obstetrics, Gynecology, Orthopedics, Pathology, Dermatology, Ophthalmology, Otology, Rhinology, Laryngology, Hygiene and other topics of interest to students and practitioners. Edited by **W. T. Longcope,** M.D. Volume III. Nineteenth series. Philadelphia and London: J. B. Lippincott Co. 1909. (Cloth, $2.00.)

The articles on treatment in this volume, three in number, are contributed by Francine, Lagane, and Waterson. The former presents the treatment of tuberculosis; Lagane deals with the present position of antitetanic serotherapy; while Waterson's article presents the singular title "Mesmer and Perkins's tractors." It is interesting to read how Mesmer reached fame through "animal magnetism" as it was termed, or "mesmerism" as it was more frequently called, and then reflect on the uses of electricity in medicine today. Mesmer was called a quack, impostor, a second Cagliostro, and his electric cures were condemned as a compound of imagination and sensuality. Waterson tells us, too, that Perkins, who invented metallic instruments, which were called tractors, for the cure of rheumatism, gout, erysipelas, and some other diseases, was expelled from the medical society of the state of Connecticut, on the ground that the instruments were patented.

The volume contains, also, five articles on medicine, four on surgery, two on gynecology and obstetrics, one on orthopedics, two on pediatrics, one on radiography, one on otology, two on neurology, one on ophthalmology, and one on pathology. A number of illustrations,—plates, plain and colored, and figures,—are introduced here and there to explain the text more fully, all contributing to make this one of the better books of the series.

---

**A Textbook of Practical Therapeutics.** By **Hobart Amory Hare,** M.D., Professor of Therapeutics in the Jefferson Medical College of Philadelphia. Thirteenth edition. Octavo, 951 pages, with 122 engravings, and 4 full-page colored plates. Lea & Febiger, Philadelphia and New York. 1909. (Cloth, $4.00; leather, $5.00, net; half morocco, $5.50, net.)

Seldom does an author have an opportunity to revise his work twelve times, as in the present instance. It is a subject, however, that often requires revision owing to the frequency with which new methods of treatment are offered. Not only this but many of the older methods are constantly being modified or are becoming obsolete, hence frequent editions become necessary to keep such a work abreast of the period. Hare in this edition considers almost every therapeutic measure of value, and places each in its appropriate place in the treatment of disease. The first part is assigned to general therapeutic considerations and it would be well for every practising physician to read this section with due care. Also what the author says regarding the newer drugs deserves considerate attention. As a practical guide to the proper administration of remedies the book has no superior.

---

**Minor and Operative Surgery,** Including Bandaging. By **Henry R. Wharton,** M. D., Professor of Clinical Surgery in the Woman's Medical College, Philadelphia. Seventh edition. 12mo, 674 pages, with 555 illustrations. Lea & Febiger, Philadelphia and New York. 1909. (Cloth, $3.00, net.)

A work so well approved as this needs but few words from us. It is divided into eight parts, the first being taken up exclusively with bandaging. The others deal with minor surgery in its several phases, including asepsis and antisepsis, fractures, dislocations, operations, amputations, excisions, resections and special operations. This edition presents the work in thorough revision in all its parts. Obsolete material has been eliminated, considerable new matter has been added, including a number of illustrations; recent minor surgical procedures have been described and the entire book has been made to present the several topics in the most modern light.

---

**Systemic (including Special) Pathology.** By **J. George Adami,** M.D., and **Albert G. Nicholls,** M.A., M.D., F.R.S., Assistant Professor of Pathology in McGill University. In one octavo volume of 1082 pages, with 310 engravings and 15 colored plates. Lea & Febiger, Philadelphia and New York. 1909. (Cloth, $6.00, net.)

In the first volume of this remarkable work, the authors dealt with the causes of disease and the morbid and reactive processes. We gave our opinion of that volume in the issue of the JOURNAL for February, 1909. Not only did we say that Adami has established his reputation as being the greatest English speaking pathologist of the day, but that judging by the first volume, this treatise would take first place as a textbook in American medical schools. After an examination of this volume we are prepared to reaffirm our then expressed opinion of author and treatise.

This, the second volume, concerns itself with the results of disease as it affects the different systems and, through them, the body as a whole. In other words, this volume covers the entire field of the individual diseases, telling us at the same time the influence of disease upon the functions of organs. To understand a disease one must familiarise himself with its pathology, must know the wreckage it leaves in its trail. In this way, through this knowledge, alone, can the proper treatment be instituted. Besides being a scientific work the two volumes possess merit for their rhetorical finish. It is a pleasure to read books written in such smooth English, in such excellent literary fashion, as the authors employ to adorn their work. A number of engravings and plates have been inserted to explain certain intricate morbid processes which prove very serviceable in elucidating the text. It is already conceded that this is the foremost American treatise on pathology, taking rank with the highest German authorities on the subject.

---

**The Principles of Hygiene.** By **D. H. Bergey,** Assistant Professor of Bacteriology, University of Pennsylvania. Third edition. Illustrated. Octavo, pp. 555. Philadelphia and London: W. B. Saunders Co. (Price, $3.00.)

In the preface to the first edition (1901), the author informed us that the purpose of his book was to aid students of medicine in the acquirement of knowledge of the principles on which modern hygienic practises are based; to aid students of architecture in comprehending the sanitary requirements in ventilation, heating, water supply, and sewage disposal; and to aid physicians and health officers in familiarising themselves with the advances recently made in hygienic practises. He has accomplished all these and in discussing the subject he tells us of food and dieting, how to exercise properly and what clothing to wear; also instructs us in personal hygiene, industrial hygiene; treats of school, military and naval hygiene; then of soil, habitations, of the vital causes of disease; of disinfection, quarantine and vital statistics. An appendix is added treating of a miscellaneous group of topics. The book has been thoroughly revised and this edition is brought down to date.

**Medical Diet Charts.** Prepared by **H. D. Arnold,** M.D., Professor of Clinical Medicine at Tufts Medical College, Boston. Philadelphia and London: W. B. Saunders Company. 1909. Single Charts, 5 cents; 50 Charts, $2.00; 500 Charts, $18.00; 1,000 Charts, $30.00.

These diet charts are most excellent indeed, and the general practitioner will find them essential in regulating scientifically the diet of his patients, and that with comparative ease. They give on one sheet all the data necessary for arranging and calculating the nutritive value of a diet and furnish a convenient means of recording the results. Chart A has space for recording the diet for five successive days or for five changes of diet for any interval of time. Chart B fulfils the same functions for a single day. There is one feature that makes these charts extremely practical—namely, the information they give as to the nutritive value of food in measures such as are used in every day life. They will prove useful to the general practitioner who has always wanted a chart of this kind.

**The Medical Record Visiting List or Physicians' Diary for 1910.** New revised edition. New York: William Wood & Company, Medical Publishers.

Whoever once has used a diary like this will find it difficult to get along without it. Year by year it will be wanted and files should be kept for future reference, as these visiting lists become a valuable historic record. This edition has been revised and contains every important feature as will be seen by the following contents.

Calendar, estimation of the probable duration of pregnancy. approximate equivalents of temperature, weight, capacity, measure, and the like, maximum adult doses by the mouth in apothecaries' and decimal measures, drops in a fluid dram, solutions for subcutaneous injection, solutions in water for atomisation and inhalation, miscellaneous facts, emergencies, surgical antisepsis, disinfection, dentition, table of signs, visiting list with special memoranda, consultation practice, obstetric engagements, record of obstetrical practice, record of vaccination, register of deaths, nurses' addresses, addresses of patients and others, cash account.

The prices of regular lists are, for sixty patients a week, with or without dates, handsomely selected red or black, morocco binding $1.50; for 30 patients a week with or without dates, same style, $1.25. These visiting lists also are fitted into genuine seal and calfskin wallets, making handsome books. The lists proper are in two books of six months each, and are removable from the wallets. They are thus much less bulky for the pocket. They are also economical inasmuch as the leather covers may be made to do service for several years. Orders should be placed at an early day. These publishers are noted for the high class medical work they send out, and these visiting lists are no exception to the rule.

---

**The Physician's Visiting List** (Lindsay and Blakiston's), for 1910. Fifty-ninth year of its publication. Philadelphia: P. Blakiston's Son & Co. (successors to Lindsay and Blakiston). (Price $1.00; 25 patients a week).

This visiting list comes to us like an old friend. We saw a copy in 1857, in the hands of a friend and have been more or less familiar with the book since that time. The annual revisions serve to keep it fresh and well up to the period of its date. This book, among other useful things, contains a calendar for 1910-1911; an obstetrical table; table of signs; an article on incompatibility; a table on the immediate treatment of poisoning; a statement on the metric system; table for converting apothecaries' weights and measures into grams; dose table; an article on asphyxia and apnea; and a comparison of clinical thermometers. The book before us is arranged for 25 patients a week but larger ones are made, running up to one hundred a week, the prices ranging from $1.00 to $2.25. Perpetual and monthly editions are also issued, bringing the book within the desire of every physician, no matter what his requirement. Moreover, the book is compact, durable, and handsome.

**Physical Diagnosis.** By **Richard C. Cabot** Assistant Professor of Medicine in Harvard University. Fourth edition. 12mo, pp. 579. Illustrated. New York: William Wood & Co. (Cloth, $3.00.)

For some years, that is to say since the first edition was issued (1905), this work has been a favorite in the field of physical diagnosis. One need not go far to ascertain the reasons for this favoritism. In the first place it is concise; second, it is based on a rational method; and, third, it is accurate. Cabot's method of teaching appeals to the seeker for knowledge because he learns something right away and it stays with him. No words are wasted with non-essentials. But we need not dwell on these features that have been pointed out already, in notices of former editions. Some slight changes have been made in this edition, the most important being "the use of the free ear in auscultation, the discussion of bronchiectasis, and the differences between the two sides of the normal chest." It is one of the most satisfactory of all the works on physical diagnosis yet published.

**Modern Materia Medica and Therapeutics.** By **A. A. Stevens,** Professor of Therapeutics and Clinical Medicine in the Women's Medical College of Pennsylvania. Fifth edition. Octavo, pp. 675. Philadelphia and London: W. B. Saunders Co. (Cloth, $3.50.)

When the first edition of this work was issued we were impressed with its value, and each new edition has increased our favorable impression created at first. It has maintained its standing as a leading textbook since 1894, when it was first put forth. The author is a well known teacher as well as writer, hence is aware of the needs of the student, and has written his book with a view to supplying those requirements. This edition has been revised in many respects, meeting the everchanging nature of an unfinished science, modifications, eliminations and additions becoming necessary to meet present conditions. The restless demand for new drugs and new methods has been met and the book is as complete as it is possible to make it.

**A Textbook of Surgical Diagnosis.** For students and practitioners. By **Edward Martin,** M.D., Professor of Clinical Surgery, University of Pennsylvania. Philadelphia. Octavo, 764 pages, with 455 engravings, largely original, and 18 full-page plates. Lea & Febiger, Philadelphia and New York. (Cloth, $5.50, net.)

The be-all and end-all of every method of treatment, especially surgical treatment, is the cure of the patient. The cure of the patient to a very large degree, must depend upon accuracy in diagnosis. Martin makes this truism, tritely stated though it may be, the basis of his treatise. In the elaboration of his scheme he has called to his aid a number of specialists—Longcope in laboratory methods, Henry K. Pancoast in *x*-ray diagnosis, Anspach in gynecologic diagnosis, Weisenburg in diagnosis of nervous affections, and de Schweinitz on the affections of the eye.

The book is written with a view not only to benefit the surgeon, but also the general practitioner and even the student. It is comprehensive but at the same time there is no waste of words, often being epigrammatic, always terse. Martin has long been distinguished in his teachings as a champion of accuracy in diagnosis, in order to lay a foundation for correct treatment. It is not surprising, therefore, to find him reducing his convictions to type and putting them out in book form. A judicious employment of illustrations has been made, and only those which distinctly portray some lesion outlined in the text are used, excepting where anatomical relations are shown. The work will add distinctly to the valuable contributions of the year to medical literature. It is one, also, that will be selected most likely by the leading colleges as a textbook.

---

**Diagnostic Methods.** Chemical, Bacteriological and Microscopical. By **Ralph W. Webster,** Assistant Professor of Pharmacological Therapeutics and Instructor in Medicine in Rush Medical College, University of Chicago. Illustrated. Octavo, pp. 641. Philadelphia: P. Blakiston's Son & Co. (Cloth, $6.00.)

Laboratory methods in diagnosis are attracting constantly more and more attention. Webster has put forth the most pretentious work yet issued on this subject. It deals with all the secretions and excretions of the body that have any practical diagnostic importance. First comes the sputum as a matter of course, then the oral, nasal, aural, and conjunctival secretions; then in the third chapter the gastric contents are dealt with. The feces becomes the subject of chapter four; parasites are considered in the fifth chapter; the urine is dealt with in chapter six, which consists of over 200 pages; secretions of the genital organs constitutes the subject of a short chapter; the blood then occupies a chapter of over 200 pages; transudates and exudates are dealt with in the next chapter; and, finally, in chapter ten, the secretion of the mammary glands is considered. From this enumeration of the general topics dealt with, the comprehensive character of the work will be noted. The thoroughness with which Webster handles a subject may be comprehended by a glance at his chapter on the urine. As we have noted already more than 200 pages are filled in detailing methods of diagnosis through examination of this excretion. The heads under which it is considered are six, while the subheads are more than 260,—showing the minute and exhaustive treatment of this topic. A similar method in dealing with the blood may be noted; and even so all through the book, the same painstaking may be observed.

Illustrations explanatory of the text are made use of here and there both in color and in black, and the printed pages are set in clear faced type, agreeable to the eye. The book is one that appeals to every studious mind and will become, doubtless, a favorite with medical teachers, students, and practitioners.

---

**Medical Gynecology.** By **Samuel Wyllis Bandler,** Fellow of the American Association of Obstetricians and Gynecologists, Adjunct Professor of Diseases of Women, N. Y. Post-Graduate Medical School and Hospital. Second edition. With original illustrations. Octavo, pp. 698. Philadelphia and London: W. B. Saunders Co. (Cloth, $5.00.)

This work by one of the cleverest of our younger gynecologists met with a full measure of success from the very beginning, and, we should add, most deservedly so. It was first issued in June, 1908, was reprinted in December of the same year, and now we have the second edition to notice in these columns. So short a time has elapsed since the book came from the press the first time, that very little new of value has appeared that merits incorporation in this edition. Some of the chapters, however, have been enlarged, notably those on electricity and hydrotherapy. The Head zones, too, have been enlarged by several pages, and additions have been made here and there throughout the book. As it now stands, it is the most complete work of its kind by an American author, and is a credit to recent gynecological literature, being distinctly an addition thereto.

